# Biomarker of early neurological deterioration in minor stroke and proximal large vessel occlusion: A pilot study

**DOI:** 10.3389/fneur.2022.1019530

**Published:** 2022-10-25

**Authors:** Zhiqiang Wang, Shuai Wang, Yuxia Li, Rongyu Wang, Lianyan Jiang, Bo Zheng, Yaodan Zhang, Qingsong Wang, Jian Wang

**Affiliations:** ^1^Department of Neurology, Hospital of Chengdu University of Traditional Chinese Medicine, Chengdu, China; ^2^Department of Neurology, Chengdu BOE Hospital, Chengdu, China; ^3^Department of Outpatient, The General Hospital of Western Theater Command, Chengdu, China; ^4^Department of Neurology, Ya'an People's Hospital, Ya'an, China; ^5^Department of Neurology, The General Hospital of Western Theater Command, Chengdu, China

**Keywords:** biomarkers, early neurological deterioration, large vessel occlusion, minor stroke, neurofilament light

## Abstract

**Background:**

Plasma neurofilament light chain (pNFL) represents one of the scaffolding proteins of central nervous system axonal injury. The aim of this study was to evaluate pNFL as a predictive biomarker for early neurological deterioration (END) in medically managed patients with large vessel occlusion (LVO) and mild presentation (NIHSS < 6).

**Methods:**

This retrospective study was developed from a prospectively collected stroke database, which was conducted at a large academic comprehensive stroke center in western China. Patients who first presented with acute ischemic stroke (AIS) within 24 h of symptom onset were continuously included. Stroke severity was analyzed at admission using the NIHSS score. The pNFL drawn on admission was analyzed with a novel ultrasensitive single-molecule array.

**Results:**

Thirty-nine consecutive patients were included in the analysis, and 19 (48.72%) patients experienced END. Patients who experienced END had significantly higher pNFL levels (mean, 65.20 vs. 48.28 pg/mL; *P* < 0.001) and larger infarct volume (mean, 15.46 vs. 9.56 mL; *P* < 0.001). pNFL was valuable for the prediction of END (OR, 1.170; 95% CI, 1.049–1.306; *P* = 0.005), even after adjusted for age and sex (OR, 1.178; 95% CI, 1.038–1.323; *P* = 0.006), blood sampling time, baseline NIHSS, TOAST classification, and infarct volume (OR, 1.168; 95% CI, 1.034–1.320; *P* = 0.012). The area under the ROC curve was 85.0% (95% CI, 0.731–0.970; *P* < 0.001). The sensitivity was 73.7%, and the specificity was 80%.

**Conclusion:**

END in minor stroke with LVO was distinguishable from those without END following the determination of pNFL in the blood samples within 24 h of onset. The pNFL is a promising biomarker of END in minor stroke with LVO.

**Clinical trial registration:**

ChiCTR1800020330.

## Introduction

Minor stroke is the most common and may represent up to 50% of cases of acute ischemic stroke (1). Proximal large vessel occlusion (LVO) is present in up to 30% of minor strokes (2). Mechanical thrombectomy is the standard of care for patients with LVO presenting with severe symptoms; however, little is known about the best treatment for patients with LVO and mild symptoms. On the one hand, most patients with LVO strokes and mild symptoms have good clinical outcomes; on the other hand, among patients with early neurological deterioration (END), 77% were dead or dependent at 3 months (3). The safety and effectiveness of endovascular therapy have been confirmed by a large number of literature (4–8). Therefore, endovascular therapy should not be given to all patients for LVO, nor should it be stopped because of mild stroke. The higher risk individuals with acute neurological deterioration are the people who need endovascular therapy. It is not difficult to see that the problem now is how to accurately predict END in this population (9).

Neurofilament light chain (NFL), as a protein exclusively expressed in neurons (10), might be a suitable candidate for this purpose because of its potential application prospects in patient monitoring, observation, and intervention research (11). The higher NFL level was found in a TIA patient who developed an ischemic stroke 1 day after blood sampling (12). It might suggest that NFL releasing in ischemic brain injury may have already started before symptoms became clinically apparent.

In light of this, this study aims to investigate the correlation between plasma NFL and END in minor stroke with LVO. We hypothesized that NFL measured within 24 h predicts END in minor stroke with LVO.

## Patients and methods

### Participants

Data are available on request from the corresponding author upon reasonable request. The study was conducted according to the principles expressed in the Declaration of Helsinki. The ethics committee of General Hospital of Western Theater Command approved sample collection and analysis (No. 2018ky06). All patients or their welfare guardians provided written informed consent for the collection of data, blood samples, and subsequent analyses. This was a single-center retrospective analysis of consecutive patients presenting with mild stroke (National Institute of Health Stroke Scale [NIHSS] < 6) and anterior circulation LVO [internal carotid artery (ICA), M1/M2 segment of the middle cerebral artery (MCA), and A1/A2 segment of the anterior cerebral artery (ACA)] from a prospectively collected stroke database (ChiCTR1800020330) (13). From 1 July 2017 to 31 December 2019, acute ischemic stroke (AIS) patients over 18 years old who first presented with stroke symptoms and were confirmed by magnetic resonance imaging (MRI) or computed tomography (CT) were collected into the database. Patients were excluded if they were treated by immediate endovascular therapy or intravenous thrombolysis before END but including those who eventually received rescue thrombectomy because of END. Therefore, rescue thrombectomy refers to the thrombectomy taken when patients have END (NIHSS increased by ≥ four points) and disabling clinical symptoms. LVO was determined by reviewing each initial computerized tomography angiography (CTA), magnetic resonance angiography (MRA), or digital subtraction angiography (DSA) report. Early neurological deterioration (END) was defined as four or more points' deterioration on NIHSS score within the first 24 h without parenchymal hemorrhage on follow-up imaging or another identified cause.

To evaluate disease severity, patients were scored by the National Institutes of Health Stroke Scale (NIHSS) score and infarct volume (calculated by MRI-DWI), referring to the previous study protocol (14).

### Blood sampling and biomarker measurement

Approximately 8 mL venous blood was collected in glass tubes containing sodium ethylenediaminetetraacetic acid (EDTA) from each subject on admission, and the time from stroke onset to blood collected was recorded. The blood samples were centrifuged at 2,000 × g at 4°C for 10 min within ~40 min of collection. Plasma supernatant was collected, divided into aliquots, and frozen at −80°C until further use. We measured pNFL by the SIMOA platform (Quanterix, Lexington, MA, USA) as described (13, 15). An in-house pool was used as an internal control and included in each assay for evaluating the assay performance. More detailed information on experimental methods can refer to our previous research (13).

### Statistical analysis

Data are presented as mean (±SD), median (interquartile range [IQR]), or numbers with percentages. For univariate analysis, the Mann–Whitney U-test, Student's *t*-test, or the chi-square test were used, as appropriate. The association of pNfL levels with END was analyzed by multiple logistic regressions and adjusted for established predictors. Criteria for the entry of variables in the regression analyses were set at *P* < 0.05, together with other clinically significant variables. To assess the diagnostic accuracy of pNfL for discriminating END and Non-END, we calculated the area under the receiver operating characteristic (ROC) curve. The optimal cutoff level for dichotomizing values was selected as the situation maximizing the Youden index. All analyses were performed using SPSS 26 (IBM, Chicago, IL). Two-tailed *P* < 0.05 was considered significant.

## Results

**Figure 1:**
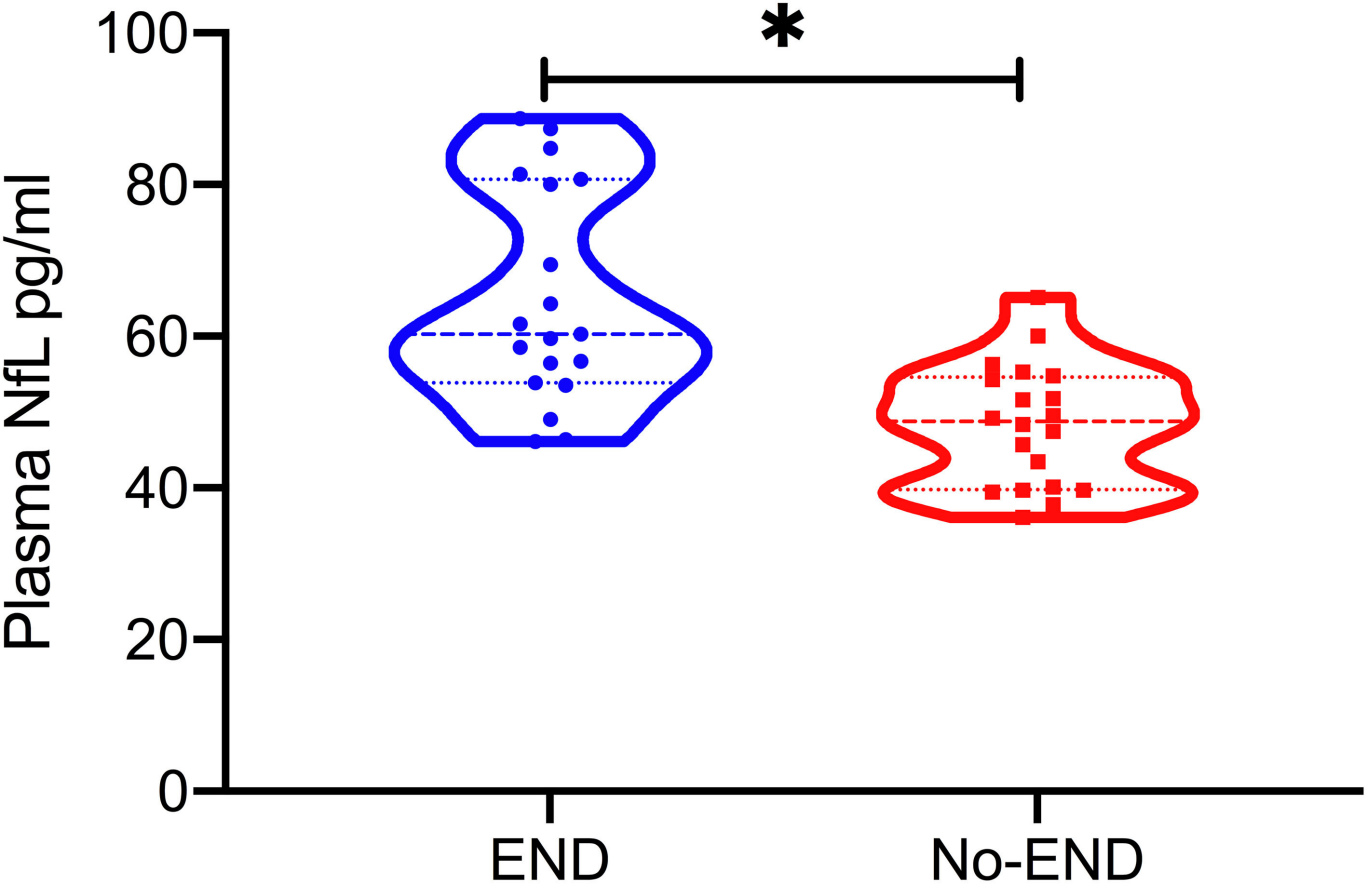
Plasma neurofilament light chain (pNFL) concentration in the diagnostic groups is shown as scatterplots. The pNFL concentration was higher in the END group compared with the Non-END group (*P < 0.001).

A total of 39 patients with acute LVO presenting with mild symptoms were included in this study. Among them, 19 (48.72%) patients experienced END. The mean age was 64.23(±10.73) years; 48.72% were men; the mean pNFL was 56.53(±14.22); and median clinical severity was three points on the NIHSS (IQR, 2–4). In all cases, the mechanism of END was progressive stroke in the same vascular territory. Baseline demographics and clinical characteristics are shown in Table 1. We found no statistical difference in the baseline characteristics between the groups with or without END, except for infarct volume and pNFL. Patients who experienced END had significantly higher pNFL levels (mean, 65.20 vs. 48.28 pg/mL; *P* < 0.001, Table 1 and Figure 1) and larger infarct volume (mean, 15.46 vs. 9.56 mL; *P* < 0.001).

**Table 1:** Demographic and clinical characteristics of the patients.

**Factors**	**Total (%)**	**END (%)**	**Non-END (%)**	***P***
Overall rate, *n* (%)	39 (100)	19 (48.72)	20 (51.28)	
Sex (male), *n* (%)	19 (48.72)	9 (47.37)	10 (50.00)	0.869
Age (y), mean (±SD)	64.23 (±10.73)	66.74 (±7.86)	61.85 (±12.65)	0.158
Vascular risk factors, *n* (%)				
Hypertension	23 (58.97)	14 (73.68)	9 (45.00)	0.069
Diabetes mellitus	12 (30.80)	5 (26.32)	7 (35.00)	0.557
Hyperlipidemia	8 (20.51)	2 (10.53)	6 (30.00)	0.132
Atrial fibrillation	8 (20.51)	2 (10.53)	6 (30.00)	0.132
Smoking	14 (35.90)	7 (36.84)	7 (35.00)	0.905
Drinking	11 (28.21)	6 (31.59)	5 (25.00)	0.648
NIHSS, median (IQR)	3 (2–4)	3 (3–4)	3 (2–4)	0.117
Infarct volume (ml), mean (±SD)	16.64 (±8.76)	15.46 (10.43–23.32)	9.56 (2.29–18.93)	**< 0.001**
TOAST classification, *n* (%)				0.208
Large-artery atherosclerosis	18 (46.15)	11 (57.89)	7 (35.00)	
Cardioembolism	10 (25.64)	5 (26.32)	5 (25.00)	
Others	11 (28.21)	3 (15.79)	8 (40.00)	
Event to blood sampling (h), median (IQR)	13.24 (±7.25)	14.37 (±7.59)	12.18 (±6.95)	0.942
Ln HbA1c (%), mean (±SD)	1.84 (±0.23)	1.83 (±0.23)	1.86 (±0.23)	0.484
Ln HsCRP (mg/L), mean (±SD)	1.25 (±0.71)	1.41 (±0.84)	1.10 (±0.54)	1.375
Plasma NfL (pg/mL), mean (±SD)	56.53 (±14.22)	65.20 (±14.29)	48.28 (±8.00)	**< 0.001**
90-day mRS (IQR)	2 (1-4)	3 (1-4)	1 (1-2)	**< 0.001**

IQR, interquartile range; NIHSS, NIH stroke scale; mRS modified Rankin Scale; pNFL, plasma neurofilament light chain concentration; TOAST, Trial of ORG 10172 in Acute Stroke Treatment.

Bold text indicates a statistically significant difference with a p-value < 0.05.

After multivariate analysis, pNFL levels (OR, 1.170; 95% CI, 1.049–1.306; *P* = 0.005) were significant for prediction of END, even after adjusted for age and sex (OR, 1.178; 95% CI, 1.038–1.323; *P* = 0.006), blood sampling time, baseline NIHSS, TOAST classification, and infarct volume (OR, 1.168; 95% CI, 1.034–1.320; *P* = 0.012; for details, see Table 2).

**Table 2:** Odds ratios for pNfL by END compared to Non-END.

**Variables**	**OR**	**95%CI**	***P***
Unadjust pNfL	1.17	1.049–1.306	**0.005**
Model 1 pNfL	1.178	1.038–1.323	**0.006**
Model 2 pNfL	1.168	1.034–1.320	**0.012**

pNFL, plasma neurofilament light chain. Model 1 adjusted for age and sex. Model 2 adjusted for Model 1 and NIHSS, TOAST classification, event to blood sampling (h), and infarct volume. Bold text indicates a statistically < 0.05.

**Figure 2:**
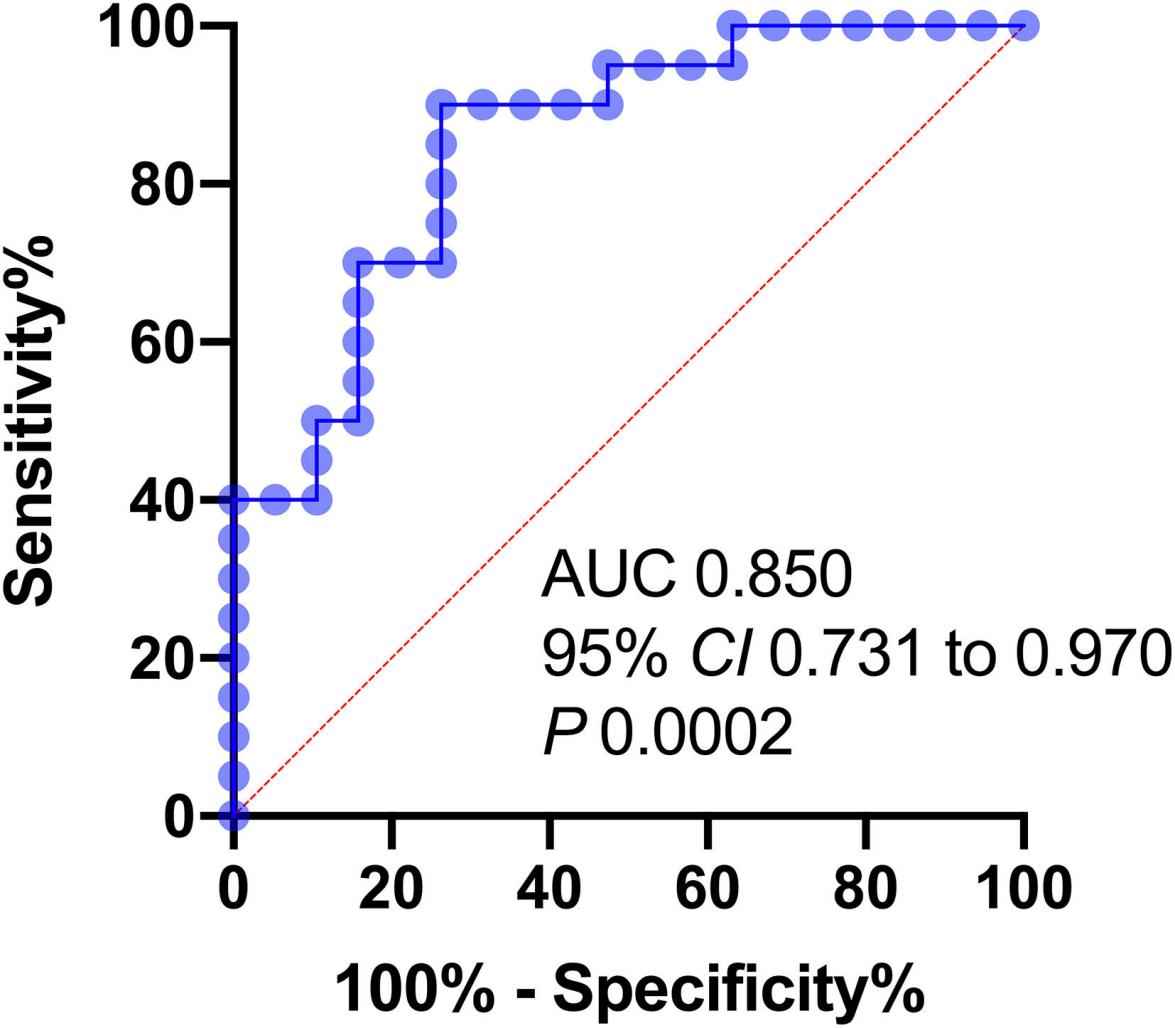
Receiver operator characteristic (ROC) curves of variables predicting END 24 h after stroke onset. AUC, area under the curve; NIHSS, NIH stroke scale; pNFL, plasma neurofilament light chain concentration.

The highest sensitivity and specificity value required to make a distinction between END and Non-END was obtained using a pNFL cutoff point 55.03 pg/mL. The area under the ROC curve was 85.0% (95% CI, 0.731–0.970; *P* < 0.001). The sensitivity was 73.7%, and the specificity was 80% (Figure 2).

## Discussion

Little is known about the best treatment for minor stroke with LVO. Endovascular therapy should not be given to all patients for LVO, nor should it be stopped because of minor stroke. The higher risk individuals with END are the people who need endovascular therapy. In the current study, pNFL levels were analyzed and quantified using a novel ultrasensitive technique in a cohort of END and Non-END patients with different etiologies. This study shows that END frequently occurs (39.4%) in patients with minor stroke and LVO. pNFL levels were shown to be elevated in patients with END compared to those with Non-END within 24 h of onset, and pNFL independently predicted END. The levels of pNFL showed significant diagnostic accuracy in discriminating patients with END from those without END. This is the first study that has investigated the pNfL levels in mild stroke with LVO.

It is unclear which factors can predict END in patients with LVO and mild symptoms. Although rescue endovascular therapy was associated with improved clinical outcomes in patients with neurological deterioration (16), primary endovascular therapy was better than secondary endovascular therapy in the case of neurological deterioration (17, 18). Accurately predicting END in this population may be helpful to select candidates for immediate transfer for additional thrombectomy. No clinical or radiological predictors of acute neurological deterioration ≥4 NIHSS points were observed on multivariable analysis, which is consistent with previous studies (16). Previous studies have indicated that admission glucose level (19), D-dimer level (20), and imaging variables (Volumes of Tmax delay) could identify patients at high risk of END following a minor stroke due to LVO (21, 22). However, these markers cannot reflect the mechanism of END. The direct pathological cause of END is neuronal damage, so looking for markers related to neuronal damage would be useful for predicting and reflecting END. Therefore, we retrospectively analyzed the stroke database of our center (13) and screened the pNFL expression of patients with mild stroke and LVO.

NFL releasing in ischemic brain injury may have already started before symptoms became clinically apparent. Previous studies have shown that pNFL expression was associated with clinical characteristics, stroke severity, and clinical outcome in stroke (11, 23–25). NFL levels also predict functional improvement in the late phase after stroke (26). Recent research results show that higher pNFL in AIS patients after endovascular therapy indicates poor outcome (27), especially the combination of pNFL and NIHSS has higher predictive value (28). These results indicate that pNFL is a specific marker of nerve injury, which can be highly expressed in various nervous system diseases (29). Interestingly, a higher pNFL level was found in a transient ischemic attack (TIA) patient who developed an ischemic stroke 1 day after blood sampling (12). In this study, there is no significant correlation between pNFL and cerebral infarction volume (assessed by DWI), which further indicates that the expression of NFL may be independent of imaging findings (the results are not shown). In addition, NFL is related to the clinical severity (30) and can distinguish different nervous system diseases (31), which indicates that the degree of neuronal damage is related to the expression of NFL and further indicates the feasibility of NFL in differentiating END. It cannot be denied that the expression of NFL also has some influencing factors. First, as mentioned above, NFL may increase based on other nervous system diseases. Therefore, the impact of other nervous system diseases must be excluded in the diagnostic process of using NFL. This study excluded patients with other possible neurological diseases and previous cerebral infarction. Second, the expression of NFL changed dynamically with time after stroke (32), so the effect of blood collection time on NFL cannot be ignored. This study showed that there was no significant difference in blood collection time between the two groups.

Given the association between END and pNFL in patients with minor stroke and acute LVO, restoration of perfusion deficits might be considered a potential treatment strategy for patients at high END risk. Since delaying endovascular therapy until neurological worsening appears to reduce its beneficial effect, immediate endovascular therapy might be considered in cases of minor stroke and LVO with a high pNfl. Further well-designed clinical trials should be conducted to prove the benefit of immediate endovascular therapy in minor stroke patients with a high risk of END.

Several limitations to this study should be noted. First, retrospective studies are prone to selection biases. In any case, prospective studies are needed to determine the value of pNFL in making triage decisions to select candidates for primary endovascular therapy. Second, the small sample size due to strict inclusion criteria raises the risk of chance findings. Third, we were limited to a cross-sectional analysis as longitudinal pNFL measurements were not available.

## Conclusion

The high expression of NFL in patients with minor stroke and proximal anterior LVO means that they are more prone to END, and these patients may benefit more from early MT treatment. This study provides objective indicators for the formulation of treatment plans for patients with minor stroke due to large vessel occlusion. As a result, further randomized controlled trials are needed to verify this association.

## Data availability statement

The raw data supporting the conclusions of this article will be made available by the authors, without undue reservation.

## Ethics statement

The studies involving human participants were reviewed and approved by Ethics Committee of General Hospital of Western Theater Command. The patients/participants provided their written informed consent to participate in this study.

## Author contributions

JW: conceptualization, methodology, supervision, and software. ZW and SW: data curation, writing the original draft, reviewing, and editing. YL, RW, and LJ: data curation and investigation. BZ and YZ: data curation. QW: conceptualization and investigation. All authors reviewed the manuscript. All authors contributed to the article and approved the submitted version.

## Funding

This study was supported by the Scientific Research Project of Health and Family Planning Commission of Sichuan Province (No. 16PJ014), Sichuan Science and Technology Department Project (Nos. 2019ZYZF0063 and 2020YJ0497), Sichuan Medical Research Project (No. S17003), and Sichuan Medical Youth Innovation Research Project (No. Q21049).

## Conflict of interest

The authors declare that the research was conducted in the absence of any commercial or financial relationships that could be construed as a potential conflict of interest.

## Publisher's note

All claims expressed in this article are solely those of the authors and do not necessarily represent those of their affiliated organizations, or those of the publisher, the editors and the reviewers. Any product that may be evaluated in this article, or claim that may be made by its manufacturer, is not guaranteed or endorsed by the publisher.
